# A Honey Trap for the Treatment of Acne: Manipulating the Follicular Microenvironment to Control *Propionibacterium acnes*

**DOI:** 10.1155/2013/679680

**Published:** 2013-05-14

**Authors:** E. Anne Eady, Alison M. Layton, Jonathan H. Cove

**Affiliations:** ^1^Department of Dermatology, Harrogate and District NHS Foundation Trust, Lancaster Park Road, Harrogate HG2 7SX, UK; ^2^Faculty of Biological Sciences, University of Leeds, Leeds LS2 9JT, UK

## Abstract

Today, as 40 years ago, we still rely on a limited number of antibiotics and benzoyl peroxide to treat inflammatory acne. An alternative way of suppressing the growth of *Propionibacterium acnes* is to target the environment in which it thrives. We conjecture that *P. acnes* colonises a relatively “extreme” habitat especially in relation to the availability of water and possibly related factors such as ionic strength and osmolarity. We hypothesise that the limiting “nutrient” within pilosebaceous follicles is water since native sebum as secreted by the sebaceous gland contains none. An aqueous component must be available within colonised follicles, and water may be a major factor determining which follicles can sustain microbial populations. One way of preventing microbial growth is to reduce the water activity (*a*_*w*_) of this component with a biocompatible solute of very high water solubility. For the method to work effectively, the solute must be small, easily diffusible, and minimally soluble in sebaceous lipids. Xylose and sucrose, which fulfil these criteria, are nonfermentable by *P. acnes* and have been used to reduce water activity and hence bacterial colonisation of wounds. A new follicularly targeted topical treatment for acne based on this approach should be well tolerated and highly effective.

## 1. Introduction

*Propionibacterium acnes* has long been implicated in the pathogenesis of inflammatory acne [1, 2]. Although its role in lesion formation is still heavily debated [3, 4], there is no doubt that treatment which reduce numbers of propionibacteria on skin are therapeutic [5, 6]. Acne is a disease of the infundibulum of the pilosebaceous follicle, and antimicrobials directed against *P. acnes* must target this site to be effective. Antibiotic therapy, now usually combined with benzoyl peroxide to minimise the risk of resistance, remains the only antimicrobial therapeutic option currently available [7]. Novel antimicrobial treatments are required that do not select for antibiotic resistance and which are more friendly to skin and household linens than benzoyl peroxide. Whilst good progress is being made with physical therapies, especially those based on light [8], development of an acne vaccine is hampered by the fact that, with a few notable exceptions, the major immunogens of *P. acnes* associated with protective responses remain poorly defined. A novel approach initially proposed by C. N. Burkhart and C. G. Burkhart [9] is to alter the microenvironment within the pilosebaceous follicle beyond the permissible boundaries for growth and survival of *P. acnes.* In principle several environmental parameters could be altered, for example, pH, oxygen tension, or ionic strength but it is our contention that one of the most promising and amenable parameters to change has so far been overlooked. We hypothesise that availability of water and possibly of one or more water soluble micronutrients limit microbial growth within follicles and that reduction of water activity represents a novel and safe therapeutic approach in acne management.

Whether the hypothesis put forward here turns out to be right or wrong does not really matter. Our aim is to stimulate more research on the follicular environment and on the attributes of *P. acnes* that make it so well adapted to exploit this rather unusual niche.

## 2. Water and Colonisation of the Pilosebaceous Follicle

It is often stated that the skin surface is a harsh environment resistant to colonisation by transient contaminating bacteria. Although recent evidence suggests that innate immune mechanisms contribute much to skin defence against microbial invaders [10], arid conditions and the “acid mantle” have long been invoked as determining factors that limit the diversity of organisms colonising the skin surface [11]. It is well documented that occlusion of the skin surface, which results in significant hydration of the skin and increase in surface pH, gives rise to a corresponding increase in the number of surface bacteria [12] even when innate defences remain intact. Similarly life for bacteria that colonise pilosebaceous follicles is likely to be stressful. As well as the lack of water, sebum is full of antimicrobial substances such as long and short chain fatty acids derived from triglycerides through the action of bacterial lipases as well as antimicrobial peptides such as defensins, cathelicidin, and histone H4 [13]. These potent inhibitors of bacterial multiplication, which can act synergistically, are produced by sebocytes in the gland and/or keratinocytes lining the duct [14, 15]. Given that *P. acnes* is susceptible to antimicrobial peptides and some sebaceous type fatty acids *in vitro*, the concentration of these substances in the sebum of colonised follicles must be subinhibitory,or the organisms must be sequestered from their effects. The pilosebaceous follicle is not a cosy environment for microbial colonisation. Compared to mucosal surfaces which support the growth of a dense and taxonomically complex resident microflora, *P. acnes* colonises a relatively “extreme” environment especially in relation to the availability of water and possibly related factors such as ionic strength and osmolarity. In adults, *P. acnes* is the predominant inhabitant of skin regions rich in sebaceous glands including the face and upper trunk [16] and in healthy skin appears to be the exclusive resident within pilosebaceous follicles [17]. In normal and acne prone skin, population densities of viable *P. acnes* within individual follicles typically exceed 10^5^, although in people with acne only a minority of follicles are colonised [18]. Presumably one or more defining characteristics of the genus *Propionibacterium* together with species specific traits make *P. acnes* particularly well adapted to the follicular environment. It is interesting to note that *Propionibacterium freudenreichii*, a dairy species that is used to manufacture Swiss hard cheeses, is also adapted to a harsh low water environment [19]. Perhaps members of this genus share an ability to grow where water is scarce or biologically unavailable.

**Figure 1:**
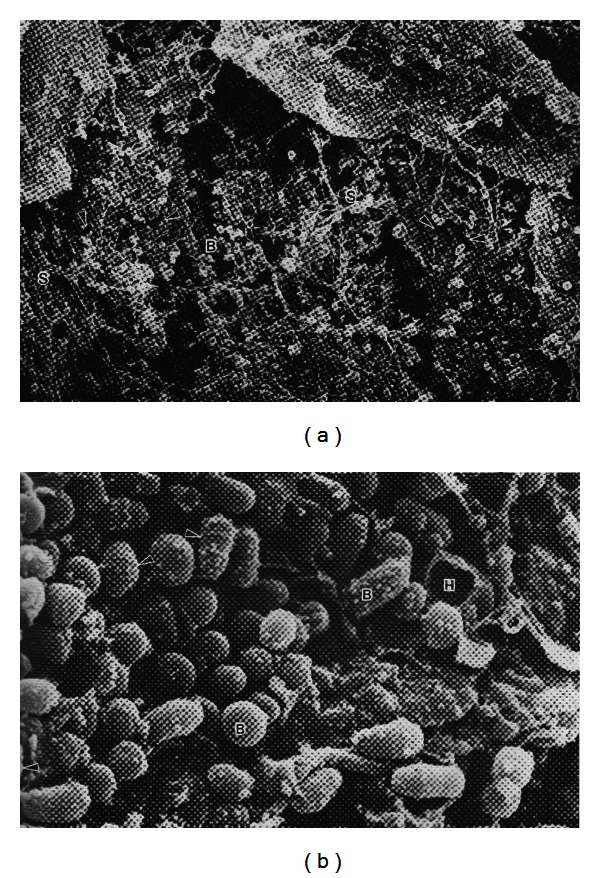
Propionibacteria in a comedone from acneic skin: interaction with sebum and corneocytes. Scanning electron micrographs of propionibacteria growing on the surface of corneocytes with an open comedone. The image on the right is a higher magnification (×15,840) and shows sebum droplets adhering to the surface of the bacterial cells. Where is the aqueous component? B: bacteria; S: sebum; H: hole in corneocyte. Arrows indicate dividing cells with visible septa. Images reproduced with permission from WH Wilborn, BM Hyde, Montes LF, Scanning Electron Microscopy of Normal and Abnormal Human Skin, 1985, VCH Publishers.

Within pilosebaceous follicles there are several possible sources of water. Water may be released along with sebaceous lipids upon rupture of mature sebocytes. Although sebum is a rich source of potential macronutrients, sebum collected from the skin surface apparently contains little or no water [20], and reported analyses of sebum do not mention the presence of water [21]. Sebum would be expected to have a higher water content if sebocytes sometimes rupture before they are fully mature. Another possibility is that water is derived exogenously, perhaps from eccrine sweat. This seems unlikely given the barrier function of skin and that exogenous water would have to permeate the follicular orifice against an outflow of hydrophobic sebum. However, studies with the asebia mouse have shown that sebaceous gland-derived glycerol contributes to stratum corneum hydration [22]. Glycerol is released from sebaceous triglycerides by the action of *P. acnes* lipases (glycerol esterases), and skin with high numbers of *P. acnes* is depleted in glycerol [23]. As a humectant, glycerol will allow sebum at the skin surface to absorb water from the atmosphere. Wertz has shown that synthetic sebum containing triglycerides but no free glycerol will take up 6% of its weight in water when equilibrated in an atmosphere saturated with water vapour [24]. Taken together, these observations suggest that sebum can retain water in the follicular lumen but do not explain where it comes from. The most likely source of unbound water is via diffusion from the dermal vasculature through the keratinocyte lining of the follicular wall. Anatomically this becomes thinner below the infundibulum in the deeper regions of the pilosebaceous follicle, and this could be a possible location for the ingress of water [25]. Unlike the exposed skin surface, evaporation is almost certainly not a significant driver of transepidermal water flux into the infundibulum, so that ingress of water would suggest a breach in the integrity of the follicle wall. The final possibility is that *P. acnes* obtains water by growing on a substratum, for example, from keratinocytes lining the infundibular wall. The wall comprises a cornified epithelium that is continuous with the stratum corneum. *P. acnes* produces a host of tissue damaging enzymes that may be able to destroy corneocyte membranes [26]. This assumption would appear to be confirmed by scanning electron micrographs of acne comedones which clearly show “holes” in shed corneocytes heavily colonised by propionibacteria (Figure 1, [27]). Shed vellus hairs present an alternative substrate, and *P. acnes* has been shown adhering to hairs within comedones [28]. Hair contains significant amounts of water [29].

## 3. Relationship between *P. acnes* Growth Rate and Rate of Sebum Secretion

The classical picture of the follicular niche is that *P. acnes* resides in a nutrient rich environment provided by a constant supply of sebaceous lipids. However, sebum almost certainly does not provide all that is necessary to support growth. Water soluble vitamins, for example, are not present in sebum and so must be provided from an aqueous compartment. *P. acnes* has an absolute requirement for pantothenate and nicotinamide and several trace elements including iron and cobalt [30, 31]. It is most unlikely that the growth limiting nutrient, which is a major determinant of the population density within a follicle, will be a sebaceous lipid. Despite this, the sebum excretion rate will exert a profound influence on the rate of growth of *P. acnes* and may determine, along with availability of water, whether a particular follicle is capable of being colonised. The sebum excretion rate shows circadian variation, and only a proportion of follicles is actively secreting sebum at any one time [32, 33]. *P. acnes* growth has to respond to these changes in sebum availability and sebum flow rate.

All bacteria have a maximum specific growth rate (*μ*_max⁡_) which they cannot exceed under any given set of environmental conditions. The growth rate will be reduced under adverse physicochemical conditions or nutrient starvation. Consequently *P. acnes* will be washed out of follicles if the rate of loss of bacterial cells due to the outward flow of sebum and desquamation of keratinocytes from the follicle wall (to which they may be attached) exceeds their ability to be replaced by growth.

It is possible to make very approximate estimations of the *in vivo* growth rate of *P. acnes* by applying chemostat theory [34] using follicular volume (*V*, expressed in litres) and the sebum flow rate (*F*, expressed in litres per hour). The rate at which the follicular content is diluted by the outflow of sebum is given by *F*/*V* = D, where Dh^−1^ is the dilution rate. Assuming that the propionibacterial population density within the follicle remains constant over time (i.e., in a steady state), the specific growth rate *μ* h^−1^ of *P. acnes* growing exponentially must match the rate of loss of cells due to dilution caused by the outflow of sebum. Thus at steady state, *μ* = D, that is, the population density of *P. acnes* doubles at the same rate, it is being diluted. Doubling time (*T*_*d*_) is also a measure of bacterial growth and is given by the expression *T*_*d*_ = ln⁡2/*μ* or 0.69/*μ*. Since D = *F*/*V* and at steady state D = *μ*, published values for the follicular volume and sebum excretion rate can be used to approximate the doubling time of *P. acnes in vivo*. The volume of pilosebaceous follicles has been reported to be 0.12–0.26 mm^3^ cm^−2^ [35] on the forehead, and typical values for the sebum excretion rate have been given as 0.25–2.8 *μ*g cm^−2^ min^−1^ at this site [36] with higher rates in subjects with acne. The measured rate is the average of the sebum output from hundreds of follicles. It is generally held to be true that only half of the follicles in a given area are actively secreting sebum at any one time [37], so that the range of values for D (equivalent to specific growth rate *μ*) is calculated to be from 0.114 h^−1^ to 3.36 h^−1^ equivalent to doubling times between 0.21 and 6.1 h. The maximum growth rate achieved by *P. acnes* under optimum laboratory conditions with an abundant supply of a fermentable carbohydrate such as glucose is 0.2 h^−1^ equivalent to a doubling time of 3.5 h. The growth rate will be reduced markedly under suboptimal physical conditions of pH, oxygen tension, and water activity and when utilising energetically less favourable carbon sources such as glycerol. For example, in the presence of oxygen (20% air saturation), *μ*_max⁡_ decreases to 0.04 h^−1^ corresponding to a doubling time of 17.25 h [38]. Even growing at its maximum specific growth rate, it would be very difficult for *P. acnes* to colonise de novo those follicles with high sebum production rates, and this may explain why most follicles in acne prone skin do not contain propionibacteria.

One piece of experimental evidence gives an indirect estimate of the actual *in vivo* growth rate of *P. acnes*. Benzoyl peroxide reduces numbers of *P. acnes* on the skin surface by an average of 2 logs (99% of baseline value). In the only study to investigate what happened after treatment, the *P. acnes* population density was observed to have partially recovered by day three and fully by day seven [39]. The doubling time calculated from the measured rate of increase is approximately 18 h. This suggests that *P. acnes* may gain entry into follicles when sebum output is low as a result of diurnal rhythm or the follicle cycle and manages to maintain a stable population by growing in contact with a substratum to which it adheres strongly (see the following).

## 4. What Is Water Activity, and How Does It Affect Microorganisms?

The term water activity is derived from fundamental principles of thermodynamics and is a measure of the energy status of water in a system. It is a measure of how efficiently the water can take part in a chemical reaction. The energy status of water can be reduced by both osmotic and matric effects. When substances such as salt (NaCl) or sugar are dissolved in water, the water activity is reduced by the number of particles in solution; hence water activity is dependent on solute concentration. For a review on life at low water activity see Grant, 2004 [40].

Essentially water activity (*a*_*w*_) = *p*/*p*_*o*_, where *p* is the vapour pressure of water in the substance and *p*_*o*_ is the vapour pressure of pure water at the same temperature. The *a*_*w*_ of pure water is 1.0. Growth of most microbes is not possible below an *a*_*w*_ of 0.85 although extremely xerophilic, and halotolerant species can grow down to an *a*_*w*_ of 0.61. Low water activity not only affects bacterial growth but also bacterial exocellular enzyme activity. Minimum *a*_*w*_ values for growth are solute dependent. When the *a*_*w*_ of the suspending medium is reduced by the dissolution of a solute such as sucrose, then water is withdrawn from microbial cells, concentrating the intracellular fluid until the internal and external values are similar. When the concentration of the intracellular fluid goes beyond a critical value, growth and metabolism cease. This is because outwardly orientated cytoplasmic pressure is the driving force for cell growth. The cells are not killed in a low *a*_*w*_ environment, but they cannot replicate.

Microbial cells have evolved ways of protecting themselves from low water activity, or so-called osmotic stress [41, 42]. Some synthesise and accumulate compatible solutes (osmoprotectants) that reduce the intracellular *a*_*w*_ with relatively minor effects on fitness such as reduced growth rates. Such solutes include proline, glutamine, betaines, glucosylglycerol, ectoine, 3-dimethylsulphoniopropionate, and some sugar alcohols such as trehalose. Accumulation of potassium ions may also occur. Although there have been no reports on this, it may be possible to turn some of these osmoprotectants against themselves by increasing the concentration outside the cell to very high levels.

## 5. Hypothesis

A new antimicrobial approach to the treatment of acne is proposed based on the hypothesis that the growth of *P. acnes* in pilosebaceous follicles is restricted by the availability of water or water soluble nutrients. It may be possible to inhibit the multiplication of propionibacteria within follicles by reducing the water activity to a level below that which permits microbial growth and colonisation of the follicle. This could be achieved by introducing a highly water soluble, lipid insoluble compound that would reach high concentrations in the aqueous fraction of the follicular contents. Suitable compounds for this purpose would be a nonmetabolisable such as sucrose or xylose or one such as ribose or fructose which is metabolisable at normal physiological concentrations. The polyol, sorbitol, is an alternative, fermented by some but not all *P. acnes* phylotypes. All except xylose have water solubilities in excess of 200%. Mixtures may be preferable to the use of a single solute. Formulation technology will need to be developed to specifically target these to follicular water via the topical route.

## 6. Evaluation of the Hypothesis and Ways of Evaluating It

### 6.1. Availability of Water in the Follicle

Although analyses have shown that sebum collected from the skin surface contains little or no water, the presence of microorganisms within normal follicles indicates that water must be present in at least those which are colonised. Similarly water can gain access to abnormal follicles, and analyses of mature noninflamed comedones (blackheads) show that the water content of these can vary from less than 10% up to 40% [43], values a lot higher than for sebum collected at the surface of healthy skin. It is reasonable to suppose that the water content of individual follicles varies in either a random or predetermined way depending upon the source. The well-known premenstrual flare that occurs in many women is associated with water retention that decreases the size of the pore [44] and may concomitantly and transiently increase the water content of the follicular lumen. Moreover, defective water barrier function in acne has been proposed [45].

Because of their depth below the skin surface, it would be technically extremely difficult to measure the level of water within follicular infundibula *in situ*. However, several experimental approaches can be used to test the idea that healthy follicles contain low levels of water. The simplest way is to collect sebum from individual follicles and analyse its water content. Sebum can be collected using a glass slide and very short skin contact to avoid coalescence of sebum drops from adjacent follicles. The skin must be precleaned first to remove skin surface film. It is also relatively straightforward to measure the water content of microcomedones extracted with cyanoacrylate glue [46] and of individual closed comedones (blackheads). It may also be possible to determine the flux of water through pilosebaceous follicles compared to stratum corneum. In order to study the dynamics of transfollicular drug delivery, a follicular closing method has been devised to occlude the follicular orifices using small drops of a varnish wax mixture leaving the stratum corneum unoccluded [47]. Transepidermal water loss (TEWL) can be measured using specific sensors [48], and it is feasible to compare TEWL of untreated skin and skin in which the follicles had been occluded. After determining the number of follicles cm^2^, it would in principle be possible to determine the mean flux of water through an individual follicle. It is anticipated that these experiments would confirm low availability of water to microorganisms within the follicle. 

So far water activity has been considered as the limiting factor for *P. acnes* growth within the follicle. In reality *P. acnes* may face greater and more complex challenges in relation to the availability of water. The lower limit of water activity to support microbial growth is 0.6. This, however, assumes that water is available in the form of a homogeneous solution which is unlikely to be the case in the hydrophobic and heterogeneous environment of the pilosebaceous follicle. The nonmiscibility of sebum and water is easily demonstrated on a microscope slide held onto the forehead to collect droplets of sebum. If a tiny drop of water is added to the slide, the sebum droplets remain discrete. In fact water-sebum interactions and the formation of droplets and hydrolipid films have been shown to depend on complex interactions between natural surfactants within the lipids that favour wetting and van der Waals forces that oppose it [49]. A discontinuous aqueous milieu would give rise to significant obstacles to mass transfer of essential nutrients for *P. acnes* and may also generate unfavourable solute concentration gradients. For example, hydrophilic salts and organic compounds could be concentrated within the aqueous phase giving rise to conditions of ionic and/or osmotic stress that must be overcome for *P. acnes* to thrive. The presence of microbial cells may influence interactions between the aqueous phase and follicular lipids. For example, hydrophobic bacteria have been shown to stabilise oil-water emulsions [50].

Meticulous anatomical and microscopic investigations are required to determine the precise location of *P. acnes* within the follicular lumen and how it interacts with available water. Processing of sections for histology typically removes water, and conventional electron micrographs appear to show follicular propionibacteria embedded in sebum [51, 52]. Water may exist within discrete droplets, and *P. acnes* may multiply only where these occur. Alternatively each bacterial cell could be surrounded by a very thin film of water and effectively inhabit a highly restrictive aqueous environment. The existence of water lipid interfaces is essential for the functioning of bacterial lipases (triglyceride esterases) on which *P. acnes* depends for acquisition of glycerol although invariably also possesses a cutinase (lidless lipase) that functions in a predominantly aqueous environment [53].

### 6.2. Physiology and Genomics of *P. acnes*

The growth of *P. acnes* within the pilosebaceous follicle may be challenged not only by the availability of water required for cellular functions but also by additional osmotic and ionic stresses. In these respects it can be argued that *P. acnes* occupies a relatively extreme environment. This idea is not as farfetched as it may seem. As reported for *Propionibacterium freudenreichii*, a related organism with GRAS status used in the manufacture of Swiss cheese, *P. acnes* has within its genome capability for synthesis and utilisation of polyphosphate and glycogen [54], characteristics of bacteria adapted to harsh environments [55]. Polyphosphates act as energy stores and enable the organism to survive stationary phase and possibly extended periods of extremely slow growth. *P. acnes* also possesses sodium and potassium transport systems similar to those found in halophiles that enable it to colonise environments of high ionic strength together with at least three systems facilitating the uptake, synthesis, and/or utilization of osmoprotectants, namely, glycine betaine, proline, and trehalose. Osmoprotectants counteract the effects of high osmotic stress by maintaining turgor pressure without compromising cell functions. An aquaglyceroporin (glycerol uptake facilitator) is invariably present within the *P. acnes* genome enabling the uptake of water, glycerol, and other small solutes.  Table 1 summarises some of the systems encoded within the *P. acnes* genome that may enable it to survive a low water follicular environment. Of note is that the genes encoding a small conductance mechanosensitive ion channel which responds to membrane stretch, the glycerol uptake facilitator, and an Osm-like protein induced by salt shock with a peroxiredoxin type function, are located close together in the genome. Of course the presence of these genes is not particularly revealing in itself; much more important is uncovering how they are regulated. By hydrolyzing sebum triglycerides, *P. acnes* lipases may create a microniche in which the organism can survive and multiply using glycerol as an energy source, osmoprotectant, and water gatherer.

In recent years there has been much interest in the ability of *P. acnes* to produce biofilms [56, 57]. Whilst it is beyond dispute that some *P. acnes* strains produce biofilms both *in vivo* and *in vitro*, the role, if any, of biofilms in the adaptation of *P. acnes* to its follicular niche and in acne pathogenesis is not understood. The chemical composition of the biofilm has not been elucidated not has its relationship with cell wall lipoglycans and possible capsular polysaccharides been established. It is plausible that cell-associated polysaccharides of *P. acnes*, whether organized into biofilms or capsules, play an important role in capturing and storing water and/or in protecting the cells from dehydration.

Gene-knockout technology can now be applied to *P. acnes* [58], and knockout mutants could be created to test the importance of different systems required for growth of *P. acnes* under conditions of low water availability. In this way the relative importance of different osmoprotectant systems, ion channels, aquaglyceroporins, and cell associated polysaccharides could be revealed.

## 7. Candidate Compounds and Formulation Technology

Compounds, mainly sugars and polyols, would be evaluated for their ability to reduce water activity below the level permissible for the growth of *P. acnes* using concentrations that can be achieved *in vivo* using an efficient follicular delivery system (Table 2). Candidate compounds must be well tolerated. There is already a precedent for using high concentrations of sugars or honey on broken skin, so the risk of intolerance when used on essentially intact skin is low. If necessary, skin tolerance tests can be performed on the formulated product. Although we have suggested that nonmetabolisable solutes should be employed, it may be possible to achieve sufficiently high concentrations of metabolisable compounds to be effective. Although there have been no reports of bacterial inhibition using compatible solvents, their use at high concentrations under conditions of low water activity has not been tested. It may well be that under these conditions even compatible solvents can be employed to reduce the water activity below a level permissible for growth. Ectoine and hydroxyectoine are very safe compounds already used on skin as humectants. They have greater affinity for water than glycerol [59]. It is intriguing to consider whether these compatible solvents could be harnessed to sequester water from *P. acnes*. Development work will need to be carried out in order to determine optimal mode of delivery of the active solute although it is anticipated that this would involve follicular targeting, for example, using encapsulation [60, 61]. Dose response studies will be required to determine the optimum concentration of solute in the formulation and to ensure that maximum concentrations of solute are achieved in the small volume of follicular water without distribution to other areas of skin. These should take into account the likely volume of the aqueous component within the lumen of pilosebaceous follicles and the range of variability from follicle to follicle and within a single follicle at different times. 

## 8. Concluding Remarks

The hypothesis proposed in this paper has been put forward to highlight the paucity of information which is currently available about the follicular niche in which *P. acnes* resides both as a commensal and as a pathogen. Almost as little is known about the bacterial attributes associated with the ability of propionibacteria to thrive in this unusual habitat. The availability of numerous *P. acnes* genome sequences and the technology to exploit them is powerless to explain the organism's role in skin health and disease unless we learn more about the environment in which it lives. More and more studies with other bacterial species are revealing that the distinction between housekeeping genes and virulence determinants is an artificial one. An example is N-acetylmuramoyl-L-alanine amidase, a hydrolase involved in septum cleavage during cell division and an autolysin, the cell wall anchoring domain of which mediates adhesion to epithelial cells in *Listeria monocytogenes* [62]. To date the focus of efforts to understand the biology of *P. acnes* has been on candidate pathogenicity determinants of the traditional kind [63, 64] not one of which has been conclusively proven to play a role in the genesis of acne lesions. It may turn out that apparently benign housekeeping genes are absolutely critical to the ability of propionibacteria to survive within follicles and these may represent better targets for the treatment of acne than virulence determinants. The use of nonirritating solutes such as sugars and polyols offers a safer alternative to the antimicrobials currently available without running the risk of selecting for antibiotic resistance and without the undesirable effects of benzoyl peroxide. There may also be medicinal product regulatory advantages to this approach. Even if water is not the growth limiting factor *in vivo*, it may still be possible in future to treat acne via niche disruption once we understand more about *P. acnes* adaptation to its ill-defined follicular habitat.

## Figures and Tables

**Table 1:** Some osmoprotectant systems detected in *P. acnes*.

Osmoprotectant	Comment
Aquaglyceroporin/glycerol uptake facilitator	Adjacent glycerol kinase suggests role of glycerol as a carbon and energy source, but glycerol-3-phosphate dehydrogenase is not in same operon.

Glycine betaine/L-proline three component ABC transporter (uptake system)	*P. acnes* can synthesise proline but not betaines or ectoine.

Trehalose synthesis	Different pathways in different strains, for example, from maltose, possibly from glycogen stores.

Osmosensitive potassium channel	Six genes; products mediate signal transduction in response to changes in turgor pressure.

Small conductance mechanosensitive ion channel Large conductance mechanosensitive ion channel	Responsive to membrane stretch such as that induced by increased turgor pressure inside the cell; small one close to glycerol uptake facilitator.

OsmC (peroxiredoxin)	Close to glycerol uptake facilitator; typically induced in late exponential phase. Strongly deregulated in *P. acnes*.

**Table 2:** Water solubilities and approximate *A*_*w*_ values for selected solutes.

Solute (MW)	Amount solute (g)	Amount water (g)	% solute	Moles/100 g	*A*_*w*_
Sucrose (342.3)	200	100	66.7	0.58	0.86
Fructose (180.2)	200	100	66.7	1.11	0.77
Mannose (180.2)	200	100	66.7	1.11	0.77
Ribose (150.1)	200	100	66.7	1.33	0.73
Sorbitol (182.2)	200	100	66.7	1.10	0.77
Maltitol (344.3)	150	100	60	0.44	0.89
Proline (115.1)	150	100	60	1.30	0.74
Rhamnose (164.2)	100	100	50	0.61	0.86
Xylose (150.1)	100	100	50	0.67	0.85
Glycerol (92.1)	100	100	50	1.09	0.77
Urea (60.1)	100	100	50	1.67	0.69
Glycine betaine (117.1)	64	100	39.0	0.55	0.87
Ectoine (142.2)	57	100	36.3	0.40	0.90
NaCl (58.4)	22	78	22	0.38	0.86

Values of *A*_*w*_ in this table were calculated using the following formula: *a*_*w*_ = 1.00/1 + 0.27 n, where n is moles of solute/100 g water.
